# Exposure to Wild Primates among HIV-infected Persons

**DOI:** 10.3201/eid1309.070338

**Published:** 2007-10

**Authors:** Matthew LeBreton, Otto Yang, Ubald Tamoufe, Eitel Mpoudi-Ngole, Judith N. Torimiro, Cyrille F. Djoko, Jean K. Carr, A. Tassy Prosser, Anne W. Rimoin, Deborah L. Birx, Donald S. Burke, Nathan D. Wolfe

**Affiliations:** * Johns Hopkins Cameroon Program, Yaoundé, Cameroon; † University of California, Los Angeles, California, USA; ‡ Army Health Research Center, Yaoundé, Cameroon; § University of Maryland Biotechnology Institute, Baltimore, Maryland, USA; ¶ Centers for Disease Control and Prevention, Atlanta, Georgia, USA; # University of Pittsburgh, Pittsburgh, Pennsylvania, USA

**Keywords:** Africa, Central, acquired immunodeficiency syndrome, HIV-1, immunocompromised host, zoonoses, dispatch

## Abstract

HIV-1 is an immunosuppressive pathogen. Our behavioral data for 191 HIV-1–infected rural Cameroonians show frequent exposure to nonhuman primates through activities such as hunting and butchering. Immunosuppression among persons exposed to body fluids of wild nonhuman primates could favor the process of adaptation and subsequent emergence of zoonotic pathogens.

Worldwide, ≈1% of the population is immunodeficient. Although immunodeficiency has numerous causes, such as malnutrition or iatrogenic medical therapies for cancer and organ transplantation, the most significant factor globally is HIV-1 infection (*1*). In 2006, ≈40 million persons were infected with HIV-1, and >50% were in sub-Saharan Africa, where AIDS caused 2.1 million deaths (*2*).

Immunodeficiency resulting from HIV-1 infection renders the host susceptible to infections usually controlled by cellular immunity through unrelenting loss of CD4+ T-helper lymphocytes. This susceptibility predisposes affected persons to common disease-causing pathogens such as *Mycobacterium tuberculosis*, *Salmonella* spp., *Coccidioides* spp., and *Histoplasma* spp. Other pathogens that are rarely pathogenic for immunocompetent persons, such as *Cytomegalovirus*, human herpesvirus-8, *Pneumocystis* spp., *Cryptococcus* spp.*,* and *M. avium* complex, also become common causes of disease.

HIV-1–induced immunosuppression has also been proposed as a factor affecting the global emergence and reemergence of diseases (*1*,*3*). Among emerging infectious diseases in humans, ≈75% are caused by zoonotic pathogens (*4*), highlighting the potentially important risk for zoonotic exposures for HIV-1–infected populations. Central African forests, where hunting and butchering nonhuman primates are common practices, provide a ripe environment for zoonotic transmission (*5*). These areas have fostered human acquisition of Ebola (*6*,*7*), monkeypox (*8*), simian immunodeficiency viruses (*9*), simian foamy viruses (*10*), and primate T-lymphotropic viruses (*11*). Because HIV-1 infection is epidemic in Africa, persons involved in hunting and butchering of wild animals (including nonhuman primates) are possibly HIV-1–infected and thus at risk for successful infection with novel zoonotic viral infections. Additionally, HIV-1–induced immunosuppression in the wider community poses an additional risk for secondary transmission that could facilitate early viral adaptation to humans (*12*).

## The Study

As part of a community-based HIV-1 prevention campaign, February 2001–January 2003, we collected oral questionnaire data about basic demographics and behavior associated with exposure to the blood or body fluids of wild animals. In addition, blood samples were collected and transported to a central laboratory for HIV testing. We present behavioral data pertaining to animal exposures of HIV-1–infected persons in 17 rural villages in Cameroon (*5*). These are key sites for the emergence of nonhuman primate retroviruses because of the high levels of human contact with wild nonhuman primates (*5*) and cross-species transmission of simian foamy virus (*10*) and primate T-lymphotropic viruses (*11*).

Study participation was voluntary and performed under a protocol approved by the Johns Hopkins Committee for Human Research, the Cameroon National Ethical Review Board and the HIV Tri-Services Secondary Review Board. A single project assurance was obtained from the Cameroonian Ministry of Health and accepted by the National Institutes of Health Office for Protection from Research Risks.

HIV testing was performed by using an ELISA/Western blot algorithm. The ORTHO HIV1/2 (ORTHO Clinical Diagnostics Gmbh, Neckargemünd, Germany) ELISA was used as the screening test, and the HIV Blot 2.2 (Genelabs Diagnostics, Singapore) Western blot assay was used for confirmation.

Complete questionnaire data and plasma samples were collected from 3,955 persons, of whom 46.3% were female and 53.7% were male. Age range was 16–97 years (42% 16–30, 27% 31–45, 21% 46–60, and 10% >60 years). Screening for HIV-1 infection found 191 seropositive persons (prevalence 4.8% overall, 1.9%–16.3% from the 17 sites), of whom 60.2% were female and 39.8% were male. No persons were HIV-2 seropositive.

**Figure 1:**
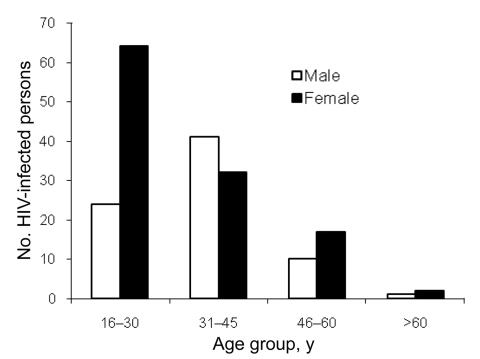
Age distribution of HIV-positive persons in 17 rural villages in Cameroon.

**Figure 2:**
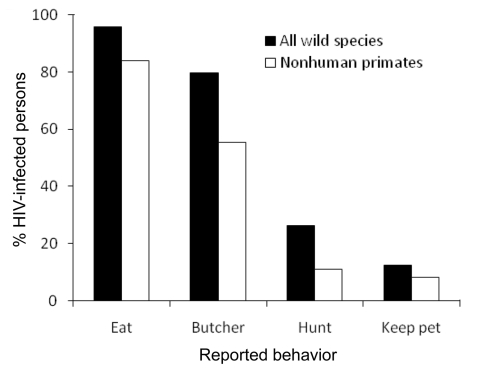
Percentage of HIV-positive persons in 17 rural villages in Cameroon who reported different types of contact with all wild animal species and with nonhuman primates.

The HIV-1–infected persons were examined in greater detail. Within the younger age group (16–30 years), women were overrepresented; among persons >30 years of age, the number of infected men and women was similar (Figure 1). Of the HIV-1–infected persons, 89.0% reported having lived in a major city or another country, compared with 82.8% of the HIV-negative study population). Agricultural activities were reported as daily activities by 46.6% of the HIV-1–positive persons; household activities, by 22.0%; and fishing, by 13.6%. Hunting was reported as a daily activity by 12.6%, and contact with wild animals was reported by an even higher proportion. Among HIV-1–positive persons, 79.6% reported butchering wild animals (as many as 20× per month), 26.2% reported hunting wild animals (also as many as 20× per month), 12.6% reported having kept a wild animal as a pet, and 95.8% reported eating wild animals (Figure 2).

HIV-1–infected persons had significant contact with nonhuman primates; hunting of these species was reported by 11.0%. Monkeys were hunted up to 10× per month (median 3× per month); chimpanzees and gorillas were hunted less frequently (always <1× per month). In terms of butchering, 55.5% reported butchering nonhuman primates: monkeys <10× per month (median 1× per month), chimpanzees 2× per month (median <1× per month), and gorillas <1× per month. Furthermore, 8.4% of HIV-1–infected persons reported keeping nonhuman primates as pets, and 83.8% reported eating nonhuman primates.

Other direct animal exposures were reported by HIV-1–infected persons (Table); bites or scratches from wild animals were reported by 12.0% and from nonhuman primates by 2.6%. Although 4.7% of persons reported having received injuries during hunting and butchering, none reported having received injuries during hunting or butchering of nonhuman primates. However, 1.7% of the rural population in this area reports such injuries (*5*).

**Table:** Injuries from wild animals received by HIV-positive persons in 17 rural villages in Cameroon, February 2001–January 2003

Participant code	Age, y	Sex	Injury	Animal
CAM2476LE	40	M	Bite or scratch	Chimpanzee
CAM4401KO	30	M	Bite or scratch	Monkey
CAM1231NG	37	M	Bite or scratch	Monkey, snake
CAM2177SA	32	F	Bite or scratch	Monkey, snake
CAM0989MO	16	F	Bite or scratch	Gorilla
CAM1188NG	19	M	Injury on finger	Not recorded
CAM2602ND	46	F	Many injuries on finger	Not recorded
CAM0212MA	45	M	Machete injury on finger during butchering	Antelope
CAM1669LE	44	M	Injury on hand during butchering	Antelope
CAM2888HA	26	F	Injuries on hand during butchering	Antelope
CAM2931HA	17	F	Injured during butchering	Antelope
CAM2162SA	30	M	Bite or scratch	Crocodile
CAM0074NY	48	M	Machete injury during butchering	Pangolin
CAM2418LE	40	M	Bite or scratch	Pangolin
CAM1788LE	24	F	Bite or scratch Injured on finger during butchering	Pangolin Snake
CAM1172NG	59	F	Bite or scratch	Rodent
CAM2387LE	33	F	Bite or scratch	Rodent
CAM3503MB	40	M	Injuries on leg during butchering	Rodent
CAM3569MB	25	M	Bite or scratch	Rodent
CAM4434KO	41	M	Bite or scratch	Rodent
CAM4225YI	38	M	Bite or scratch	Insects
CAM4233YI	22	F	Bite or scratch	Snails
CAM0908MO	39	M	Bite or scratch	Snake
CAM1590LE	31	M	Bite or scratch	Snake
CAM1970LE	28	F	Bite or scratch	Snake
CAM2190SA	65	F	Bite or scratch	Snake
CAM2345LE	30	F	Bite or scratch	Snake
CAM2378LE	52	F	Bite or scratch	Snake
CAM2973HA	40	F	Bite or scratch	Snake
CAM3674SO	32	M	Bite or scratch	Snake
CAM4020MU	32	M	Bite or scratch	Snake

## Conclusions

These data demonstrate an overlap of areas where HIV-1 is epidemic and areas where human-nonhuman primate contact is common. This overlap is cause for concern because humans and nonhuman primates share susceptibility to a range of pathogens, and the potential for successful cross-species transmission from nonhuman primates to humans is considered great (*5*). Access to treatment for HIV-1 infection is improving but is limited in remote central African communities; progressive disease and immunosuppression develop in most persons in these areas. Exposure of immunocompromised persons to nonhuman primates poses ongoing opportunities for zoonotic viruses to leap to humans, and the high concentration of other immunocompromised hosts offers an increased risk for secondary transmission and adaptation to humans. The emergence of HIV-1 is an example of such a process; data suggest several abortive nonhuman primate-to-human transmission events before eventual establishment of the HIV pandemic. This foothold gained by HIV-1 may now offer a boost for other pathogens to enter the human population. Moreover, the prevalence of HIV-1 in rural areas is lower than that in adjacent urban communities in Cameroon (*13*) and may increase. Such circumstances are not limited to central Africa; recent reports from Asia have demonstrated the risk for zoonotic infections with nonhuman primate viruses (*14*,*15*). And although nonhuman primates may present particular risks for disease emergence, HIV-associated immunosuppression likely increases the risk for acquisition, adaptation, and emergence of zoonoses infecting other animals that are hunted extensively in these communities (Figure 2), such as monkeypox and hantaviruses in rodents and Lyssavirus in bats.

The risk for emergence of novel zoonotic infections in rural hunting communities should be considered in healthcare policy. Community health education and HIV/AIDS counseling should account for the fact that many persons in these communities rely on wild animals for food and household income. Targeted interventions could include culturally appropriate suggestions for avoiding handling or butchering of wild animals, such as developing alternative food sources, or taking precautions if such activities are necessary. Reducing the prevalence of HIV-1–induced immunosuppression through prevention and treatment and minimizing zoonotic exposures will be crucial for preventing future outbreaks of novel viral pathogens in humans.
